# Bacteriophages for treating urinary tract infections in patients undergoing transurethral resection of the prostate: a randomized, placebo-controlled, double-blind clinical trial

**DOI:** 10.1186/s12894-017-0283-6

**Published:** 2017-09-26

**Authors:** Lorenz Leitner, Wilbert Sybesma, Nina Chanishvili, Marina Goderdzishvili, Archil Chkhotua, Aleksandre Ujmajuridze, Marc P. Schneider, Andrea Sartori, Ulrich Mehnert, Lucas M. Bachmann, Thomas M. Kessler

**Affiliations:** 1 Neuro-Urology, Spinal Cord Injury Center & Research, University of Zürich, Balgrist University Hospital, Zürich, Switzerland; 2 Department of Urology, University Hospital Basel, Basel, Switzerland; 3 The Eliava Institute of Bacteriophage, Microbiology, and Virology, Tbilisi, Georgia; 4 Tsulukidze National Center of Urology, Tbilisi, Georgia; 5 Medignition Inc., Research Consultants, Zürich, Switzerland

**Keywords:** Bacteriophages, Antibiotics, Urinary tract infection, Randomized placebo-controlled double-blind trial, Resistance

## Abstract

**Background:**

Urinary tract infections (UTI) are among the most prevalent microbial diseases and their financial burden on society is substantial. The continuing increase of antibiotic resistance worldwide is alarming. Thus, well-tolerated, highly effective therapeutic alternatives are urgently needed. Although there is evidence indicating that bacteriophage therapy may be effective and safe for treating UTIs, the number of investigated patients is low and there is a lack of randomized controlled trials.

**Methods and design:**

This study is the first randomized, placebo-controlled, double-blind trial investigating bacteriophages in UTI treatment. Patients planned for transurethral resection of the prostate are screened for UTIs and enrolled if in urine culture eligible microorganisms ≥10^4^ colony forming units/mL are found. Patients are randomized in a double-blind fashion to the 3 study treatment arms in a 1:1:1 ratio to receive either: a) bacteriophage (i.e. commercially available Pyo bacteriophage) solution, b) placebo solution, or c) antibiotic treatment according to the antibiotic sensitivity pattern. All treatments are intended for 7 days. No antibiotic prophylaxes will be given to the double-blinded treatment arms a) and b). As common practice, the Pyo bacteriophage cocktail is subjected to periodic adaptation cycles during the study. Urinalysis, urine culture, bladder and pain diary, and IPSS questionnaire will be completed prior to and at the end of treatment (i.e. after 7 days) or at withdrawal/drop out from the study. Patients with persistent UTIs will undergo antibiotic treatment according to antibiotic sensitivity pattern.

**Discussion:**

Based on the high lytic activity and the potential of resistance optimization by direct adaptation of bacteriophages, and considering the continuing increase of antibiotic resistance worldwide, bacteriophage therapy is a very promising treatment option for UTIs. Thus, our randomized controlled trial investigating bacteriophages for treating UTIs will provide essential insights into this potentially revolutionizing treatment option.

**Trial registration:**

This study has been registered at clinicaltrials.gov (www.clinicaltrials.gov/ct2/show/NCT03140085). April 27, 2017.

## Background

Urinary tract infections (UTI) are highly prevalent and put a substantial financial burden on the health care systems worldwide. In the USA alone, over 7 million physician consultations are due to UTIs [1], resulting in estimated direct and indirect costs of 1.6 billion US dollars [1]. Further, UTIs account for more than 100′000 hospital admissions annually [1], and for at least 40% of all hospital-acquired infections [2]. The increasing threat of antibiotic resistance, mainly due to uncritical use of antibiotics [3, 4], and the subsequent absence of access to effective antimicrobials constitutes a challenge for the future [5]. As UTIs account for approximately 15% of all community-prescribed antibiotics in the USA [6], they play an important role for direct antibiotic selection pressure. Thus, well-tolerated therapeutic alternatives to treat UTIs and to reduce antimicrobial resistances are highly warranted.

In 1917, d’Hérelle proposed the use of bacteriophages to treat bacterial infections. After a colorful episode, the discovery of penicillin by Alexander Fleming declined the interest for bacteriophages in the Western world rapidly [7]. At present, bacteriophage therapy is well accepted and registered in East European and post-Soviet countries like Georgia, Ukraine, Belarus, and Russia. Lately the use of bacteriophages as a target therapy against bacterial pathogens has gained a renewed interest. Reviews about several reports of successfully applied bacteriophage therapies for different medical specializations have been published [7, 8], and the role of bacteriophage as a possible treatment for difficult to treat microorganisms has been encountered [9–11]. A recent in vitro study could show excellent results (i.e. a high lytic activity) of commercially available bacteriophages for the most common bacterial strains found in UTIs [12]. Regarding UTIs, several current clinical studies showed positive effects of the use of bacteriophage therapy [10, 13–15]. Khawaldeh et al. reported on the success of adjunctive bacteriophage therapy after repeated failure of antibiotics alone [10]. An article in Russian language (http://www.bionow.ru/bnows-1020-2.html) describes successful treatments for topically applied (i.e. administrating bacteriophages into the urinary bladder) bacteriophages for several patients. However, well conducted clinical trials, a defined frame for bacteriophage therapy in the current Medicinal Product Regulation, and well-defined, safe bacteriophage preparations are still lacking. In consequence, the use of bacteriophage therapy is still not accepted as an official treatment against infectious diseases in the Western world [16, 17].

In line with recommendations of a multi-disciplinary expert panel on acceptance and re-implementation of bacteriophage therapy [11], we therefore designed a randomized, placebo-controlled, double-blind clinical trial to assess the efficacy and safety of intravesical bacteriophage treatment. We hypothesize that intravesical bacteriophage treatment in patients with UTIs due to *E. coli* and other uropathogens, shows a 40% increase in success rate (normalization of urine culture defined as no evidence of bacteria, i.e. <10^4^ colony forming units/mL) as compared to placebo treatment within 7 days. This difference is considered to be clinically relevant. We also hypothesize that bacteriophage treatment is non-inferior to antibiotic treatment in terms of treatment success rates, with a non-inferiority margin of 35%.

## Methods and design

### Study design

This study is a randomized, placebo-controlled, double-blind trial investigating bacteriophages in UTI treatment. The study is conducted at the Tzulukidze National Center of Urology (TNCU), Tbilisi, Georgia. Phage preparations (i.e. Pyo bacteriophage solution: commercially available and registered in Georgia) and continuous adaption cycles during the course of the study, to enhance the treatment effect, are done at the Eliava Institute of Bacteriophages, Microbiology, and Virology in Tbilisi (EIBMV), Georgia.

**Fig. 1:**
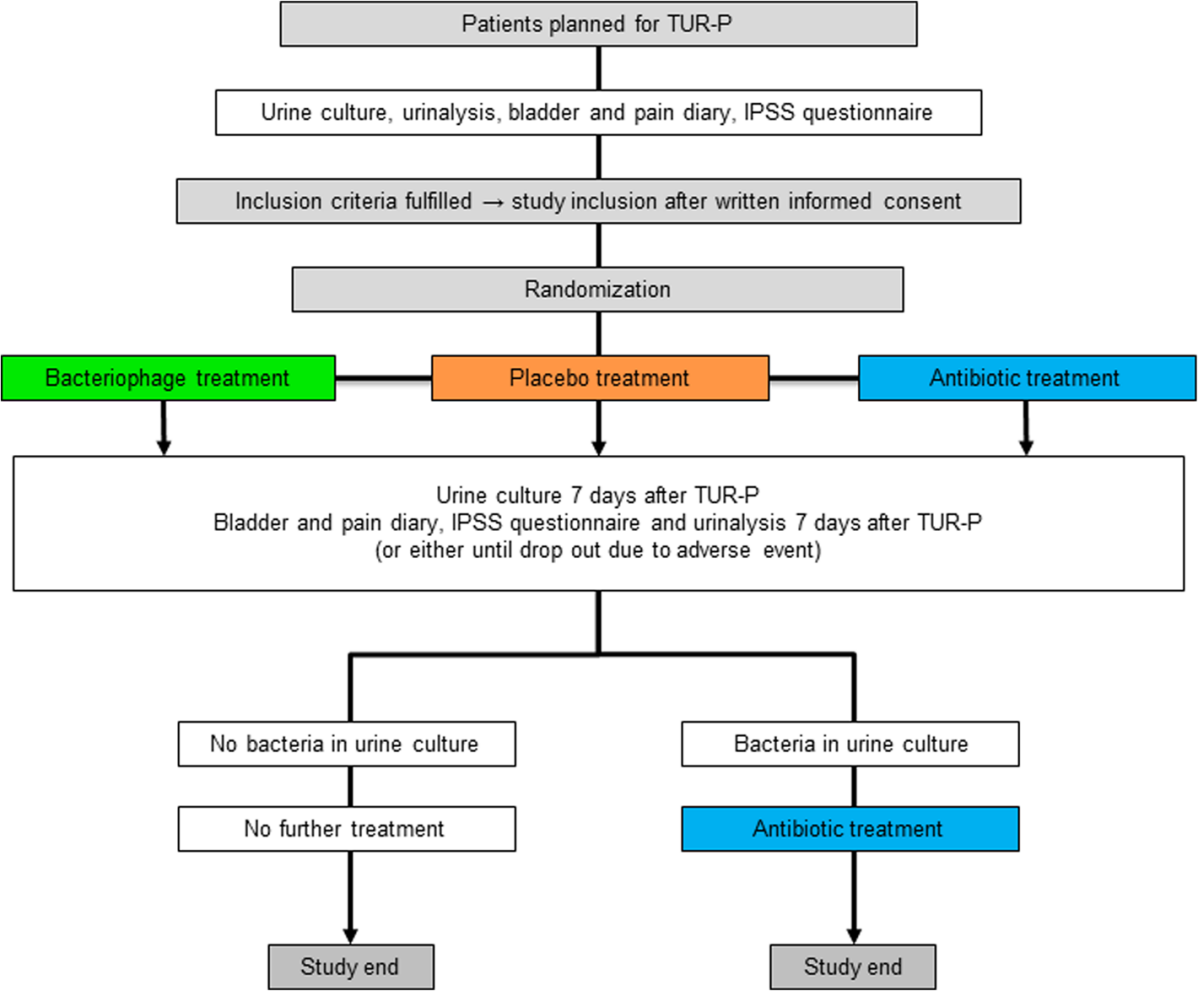
Flow chart of the procedures

**Fig. 2:**
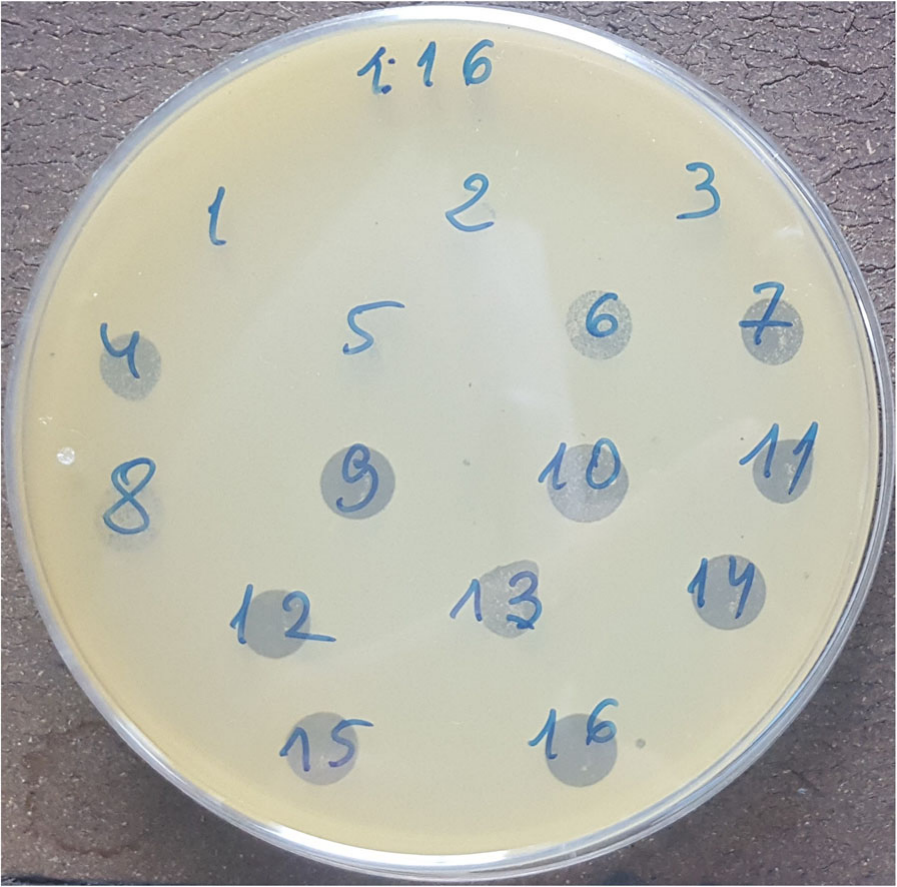
Bacteriophage sensitivity testing to *Escherichia coli*: The *Escherichia coli* culture reacts positively to 12 phages out of 16. Confluent (complete) lysis can be seen for phages #9, 14 and 16, overgrown (partial) lysis for phages #4, 6, 7, 8, 10, 11, 12, 13, 15 and 16, respectively

Note: As it is common practice when working with bacteriophage cocktails, the Pyo bacteriophage will be subjected to adaptation cycles during the course of the study.

Figure 1 gives an overview of the procedures. Patients planned for transurethral resection of the prostate are screened for UTIs at the TNCU. Urine cultures including antibiotic sensitivity testing are performed in a duplicate manner. If eligible microorganisms which would potentially match with the type of bacteriophage present in Pyo bacteriophage (5 components: Enterococcus spp., *Escherichia coli*, Proteus mirabilis, Pseudomonas aeruginosa, Staphylococcus spp., and Streptococcus spp.) are detected, urine cultures are then sent for bacteriophage sensitivity testing to the EIBMV (Fig. 2) [12]. In case of a positive in-vitro sensitivity testing, concluded within 24 h after receipt of the urine culture, patients will be asked for study participation. After written informed consent, patients are randomized in a double-blind fashion to the study treatment arms in a 1:1:1 ratio to receive either: a) Pyo bacteriophage solution, b) placebo (sterile bacteriology media with identical color as treatment arm a), or c) antibiotic treatment according to the antibiotic sensitivity pattern, respectively. Treatment arms a and b will be double-blind but treatment arm c, i.e. the control arm representing common clinical practice, is open label. All treatments are intended for 7 days starting at the day of surgery (arm c) or one day thereafter (arm a and b). Prior and at the end of treatment (i.e. after 7 days) or at withdrawal / drop out, urinalysis, urine culture, bladder and pain diary [18] and an International Prostate Symptom Score (IPSS) questionnaire [19] will be taken. Patients with persistent bacteriuria will undergo antibiotic treatment according to antibiotic sensitivity.

### Study population and recruitment

According to the inclusion and exclusion criteria (Table 1), we will investigate patients planned for transurethral resection of the prostate presenting with UTIs (defined as ≥10^4^ colony forming units/mL and symptoms such as urgency, frequency, and/or dysuria). The following variables will be considered: age, type of bladder emptying, prostate size, urinalysis, bladder and pain diary, IPSS questionnaire.

**Table 1:** Study inclusion and exclusion criteria

Inclusion criteria	Exclusion criteria
• Age > 18 years • Urine culture (taken by mid-stream urine; or from the existing transurethral or suprapubic catheter): ≥10^4^ colony forming units/mL of predefined uropathogens (i.e. Enterococcus spp., *Escherichia coli*, Proteus mirabilis, Pseudomonas aeruginosa, Staphylococcus spp., and Streptococcus spp.) • Uropathogens sensitive to Pyo bacteriophage • Symptoms: Urgency, frequency, and/or dysuria • Written informed consent	• Fever >38 °C • CRP >100 mg/L • Acute prostatitis • Concomitant fungal urinary tract infection • Current antibiotic treatment or antibiotic treatment within the last 7 days (exceptions: subjects with an active catheter associated urinary tract infection who have received prior antibiotics may be enrolled provided a minimum of 48 h has elapsed between the last dose of the prior antibiotic and the time of obtaining the baseline urine specimen. Subjects receiving current antibiotic prophylaxis for catheter associated urinary tract infection who present signs and symptoms consistent with an active new catheter associated infection may be enrolled provided all other eligibility criteria are met including obtaining a pre-treatment qualifying baseline urine culture) • Any rapidly progressing disease or immediately life-threatening illness including but not limited to: acute hepatic failure, respiratory failure, and septic shock • No informed consent

### Determination of sample size

We are planning a study of independent cases and controls with 1 control per case. Superiority of bacteriophage versus placebo treatment: We assume the success rate among controls to be 0.2. If the true success rate for experimental subjects is 0.6, we will need to study 27 experimental and 27 control subjects to be able to reject the null hypothesis that the success rates for experimental and control subjects are equal with probability (power) 0.8. The type I error probability associated with this test of this null hypothesis is 0.05.

Non-inferiority of bacteriophage versus antibiotic treatment: We assume the success rate among subjects receiving bacteriophage treatment to be 0.6 and we accept a non-inferiority margin of 0.35. If the true success rate for experimental subjects is 0.95, we will need to study 27 experimental subjects and 27 control subjects to be able to remain within the non-inferiority margin with a probability (power) 0.8. The type I error probability associated with this test of this alternative hypothesis is 0.05. We will use a continuity-corrected chi-squared statistic or Fisher’s exact test to evaluate these hypotheses.

Thus, we will include 27 patients per treatment group, i.e. 81 patients in total. Withdrawn patients will be replaced.

### Study location and partners

Tzulukidze National Center of Urology, Tbilisi, Georgia: Patient treatment.

Eliava Institute of Bacteriophages, Microbiology, and Virology in Tbilisi, Georgia: Phage preparation and adaptation.

Neuro-Urology, Spinal Cord Injury Center & Research, University of Zürich, Balgrist University Hospital, Zürich, Switzerland: Study design, monitoring and statistical support.

### Investigations

The patients planned for transurethral resection of the prostate with a positive urine culture (≥10^4^ colony forming units/mL) of predefined uropathogens sensitive to Pyo bacteriophage (i.e. Enterococcus spp., *Escherichia coli*, Proteus mirabilis, Pseudomonas aeruginosa, Staphylococcus spp., and Streptococcus spp.) and fulfilling the study inclusion criteria will be included into the study after providing written informed consent. The patients will be assessed using bladder and pain diary [18] and an IPSS questionnaire [19]. The Fig. 1 gives an overview of the procedures that patients will undergo during the study.

After inclusion, patients will be randomized in a double-blind fashion to the study treatment arms in a 1:1:1 ratio. Patients of all study arms will undergo monopolar transurethral resection of the prostate and insertion of a suprapubic catheter if not already present. Study arm a) will receive bacteriophage solution (Pyo bacteriophage) and study arm b) placebo solution (sterile bacteriology media). Either solution will consist of 20 mL and will have an identical appearance for both the bacteriophage and the placebo, respectively. An investigator not involved in the assessment of the clinical outcome will deliver the solution and teach the patient / health care provider how to instill the solution into the bladder. The solution will be instilled using the suprapubic catheter, 2 times per 24 h (i.e. 8.00, 20.00) for 7 days, starting the first day after surgery. The patients will be asked to retain the solution in the bladder for approximately 30–60 min. No antibiotic prophylaxes will be given to the study treatment arms a and b.

Study arm c) will receive an antibiotic treatment according to the antibiotic sensitivity pattern and common clinical practice.

At the end of treatment (i.e. after 7 days) or at withdrawal / drop out, urinalysis, urine culture, bladder and pain diary [18] and an IPSS questionnaire [19] will be taken. Patients with persistent UTIs will undergo antibiotic treatment according to antibiotic sensitivity for 7 days.

Drop out criteria for patients in the study arm a and b are fever >38 °C, CRP >100 mg/L, other clinical signs or symptoms for a systemic infection or withdraw from the patients. These patients will undergo antibiotic treatment according to antibiotic sensitivity.

### Safety

The investigators will inform the patients, the study monitoring board, and the ethics committee if it becomes evident that the disadvantages of participation may be significantly greater than was foreseen in the research proposal. The study will be suspended pending further review by the study monitoring board, except insofar as suspension would jeopardize the patients’ health. The investigators will take care that all patients are kept informed.

Adverse events will be assessed and categorized according to the National Cancer Institute Common Terminology Criteria for Adverse Events (CTCAE) version 4 in grade 1 to 5 (http://ctep.cancer.gov/protocolDevelopment/electronic_applications/ctc.htm). All adverse events will be followed until they have abated, or until a stable situation has been reached. Depending on the event, follow-up may require additional tests or medical procedures as indicated, and/or referral to the general physician or a medical specialist.

In the case of withdrawal of consent to participate in the study, all possible efforts will be made to convince the patient to continue to have safety follow-up evaluations.

In the event one of the following situations arises among treated patients during the conduct of the study, the study will be temporarily suspended and a comprehensive safety review conducted evaluating if the study has to be terminated prematurely:

- Any death secondary to rapid unexpected progression of an underlying medical condition.
- Severe clinical or neurological deterioration in more than one subject.
- Any other serious adverse event determined by the study monitoring board to be a reason to suspend the study.

### Study outcome measures

Primary: Success of intravesical treatment, defined as normalization of urine culture (no evidence of bacteria, i.e. <10^4^ colony forming units/mL) after 7 days of bacteriophage, placebo, or antibiotic treatment.

Secondary: Adverse events, in categorization according to the National Cancer Institute Common Terminology Criteria for Adverse Events (CTCAE) version 4 in grade 1 to 5 (http://ctep.cancer.gov/protocolDevelopment/electronic_applications/ctc.htm) during treatment phase.

Tertiary: a) Changes in bladder and pain diary assessment of number of voids, number of leakages, post void residual, pain assessment using a visual analog scale (0 (no pain) to 10 (strongest possible pain)), b) IPSS items at baseline versus day 7 under intravesical bacteriophage, placebo, or antibiotic treatment.

### Data analysis

#### Statistics

Interval scaled variates will be summarized with means and standard deviations (SD) or medians and interquartile ranges where appropriate. Dichotomous variates will be described as ratios and percentages.

#### Univariate analysis

T-tests will be used to compare means between groups and chi-squared tests to compare dichotomous variables.

#### Multivariate analysis

To adjust for unequal distribution of parameters at baseline, multivariate regression models, linear models in case of an interval scaled outcome and logistic regression in case of a dichotomous outcome will be performed.

## Discussion

UTIs are among the most prevalent microbial diseases and their financial burden on society is substantial. The continuing increase of antibiotic resistance worldwide is alarming; thus, well-tolerated, highly effective therapeutic alternatives are urgently needed. Although there is evidence indicating that bacteriophage therapy may be effective and safe for treating UTIs, the number of investigated patients is low and there is a lack of randomized controlled trials. Thus, well-designed prospective studies are urgently needed to draw definitive conclusions in the ambitious research field of UTIs. We therefore designed this first randomized, placebo-controlled, double-blind clinical trial to assess the efficacy and safety of intravesical bacteriophage for treating UTIs. This trial will significantly influence the future management of UTIs and form a basis for further studies involving bacteriophages for treating different bacterial infections. Moreover, the findings of our research project will provide further stimuli for competent authorities and physicians to use bacteriophages, as additional tools, in the prevention and treatment of otherwise virtually untreatable infections. In addition, the trial is multidisciplinary and will significantly influence all involved disciplines, i.e. urology, microbiology and infectious diseases. It will promote future multidisciplinary, multicenter approaches and collaborations further improving patients’ medical care and it will also raise the acceptance to use bacteriophages in western civilizations and accelerate regulations processes.

## Abbreviations

CTCAE: Common Terminology Criteria for Adverse Events
EIBMV: Eliava Institute of Bacteriophages, Microbiology, and Virology
IPSS: International Prostate Symptom Score
TNCU: Tzulukidze National Center of Urology
UTI: Urinary tract infections
